# Ileo-ileal knot causing acute gangrenous small bowel obstruction: a case report

**DOI:** 10.1186/s13256-024-04404-7

**Published:** 2024-02-23

**Authors:** Yohannis Derbew Molla, Melese Birara Mequanint, Solomon Hailesilassie Bisrat, Gebrehiwot Aderaw Workneh, Hirut Tesfahun Alemu

**Affiliations:** 1 Department of Surgery, University of Gondar Specialized Hospital, Gondar, Ethiopia; 2 University of Gondar Specialized Hospital, Gondar, Ethiopia

**Keywords:** Ileo-ileal knotting, Intestinal obstruction, Emergency, Case report

## Abstract

**Background:**

Ileo-ileal knotting is a very rare cause of small bowel obstruction, and only a few reports have been published. Small bowel obstruction (SBO) is one of the most common emergency surgical conditions that require urgent evaluation and treatment and is one of the leading causes of emergency surgical admission. There are many causes of SBO that are known in general surgical practice, and these causes are different in the developing and developed worlds.

**Clinical presentation:**

In this article, we present a case of acute gangrenous SBO secondary to ileo-ileal knotting in a 37-year-old Ethiopian female patient after she presented with severe abdominal cramp, vomiting, and abdominal distension of 4 hours duration. The patient was operated on intraoperatively; she had gangrenous small bowel obstruction caused by ileo-ileal knotting. Later, the patient was discharged and improved after 12 days of hospital stay.

**Conclusion:**

Ileo-ileal knotting should always be considered in the differential diagnosis of acute small-bowel obstruction. The diagnostic difficulty and the need for urgent treatment of this condition to yield optimal results are discussed.

## Introduction

Intestinal obstruction is one of the most common surgical conditions that require urgent evaluation and treatment and small bowel obstruction remains the leading cause of hospital admission worldwide. The differential diagnosis of small bowel obstruction includes adhesions, neoplasm, hernia, Crohn’s disease, and radiation Introduction [1]. An intestinal knot is a very rare cause of bowel obstruction. It was first described by Riverius in the sixteenth century and by Rokitansky in 1836 [2]. There are several types of intestinal knots which include: appendico-ileal, ileo-cecal, ileo-ileal, ceco-sigmoid and ileo-sigmoid. Of them, ileo-sigmoid is the commonest and ileo-ileal is the rarest [3]. Intestinal knots usually occur when there is free movement of the intestine with narrow peritoneal attachment. In ileo-ileal knotting one loop of ileum encircles over the other static ileum to form a knot [4]. Here, we present a rare case of gangrenous small bowel obstruction caused by ileo-ileal knotting in a 37-year-old-female who required emergency surgical intervention to save her life.

## Clinical presentation

A 37-year-old Ethiopian female presented to the emergency department with severe colicky abdominal pain of 4 h duration with associated frequent bilious vomiting and abdominal distention. She has been a known retroviral-infected patient on HAART for the past 6 years with an unknown baseline CD4 count and viral load. She has no history of previous surgery, trauma, or tuberculosis treatment.

Upon examination, she was acutely sick, looking in intermittent painful distress. Her vital signs were a pulse rate of 100 beats per minute, a blood pressure of 90/70 mmHg, a respiratory rate of 32/min, and a temperature of 36.5 °C. She has a dry tongue and buccal mucosa. The abdomen was slightly distended with generalized tenderness, guarding, and rigidity. She had a hypoactive bowel sound. On digital rectal examination, there was stool on the examining finger, and no abnormality was detected. The remainder of the examinations were unremarkable.

**Fig. 1:**
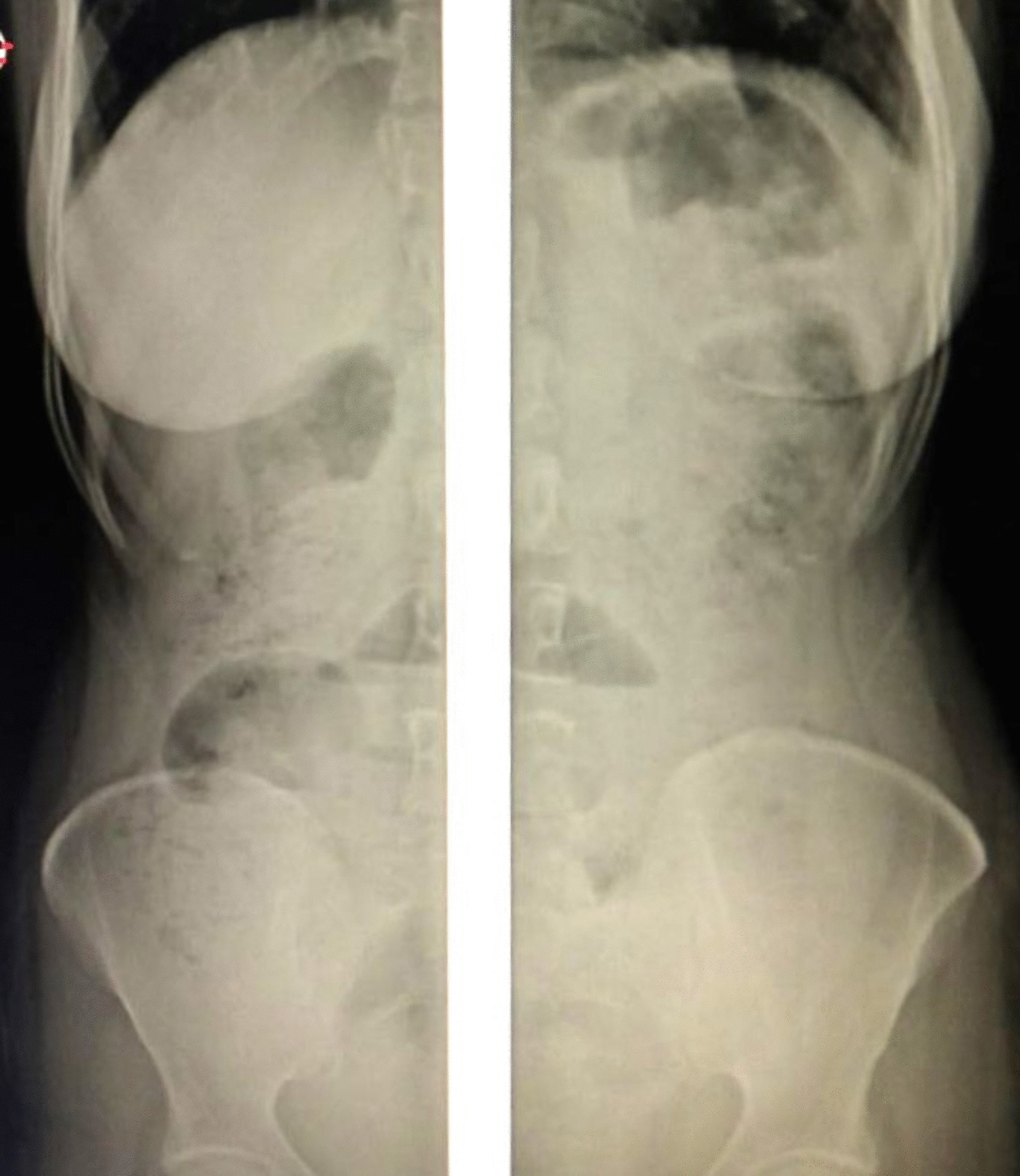
preoperative X-ray showing dilated small bowel loops with air-fluid level

On investigations, laboratory results showed leukocytosis of 18,400 with 87.7% neutrophils, hemoglobin of 14.1 g/dl, and a platelet count of 329,000/microliter of blood. Organ function tests and serum electrolytes were in the normal range. Abdominal ultrasound showed multiple dilated small bowel loops and intra-abdominal fluid collection in para-vesical space and the Morrison’s pouch with a maximum depth of 4 cm. A plain abdominal X-ray showed dilated small bowel loops with few centrally located air-fluid levels (Fig. 1).

**Fig. 2:**
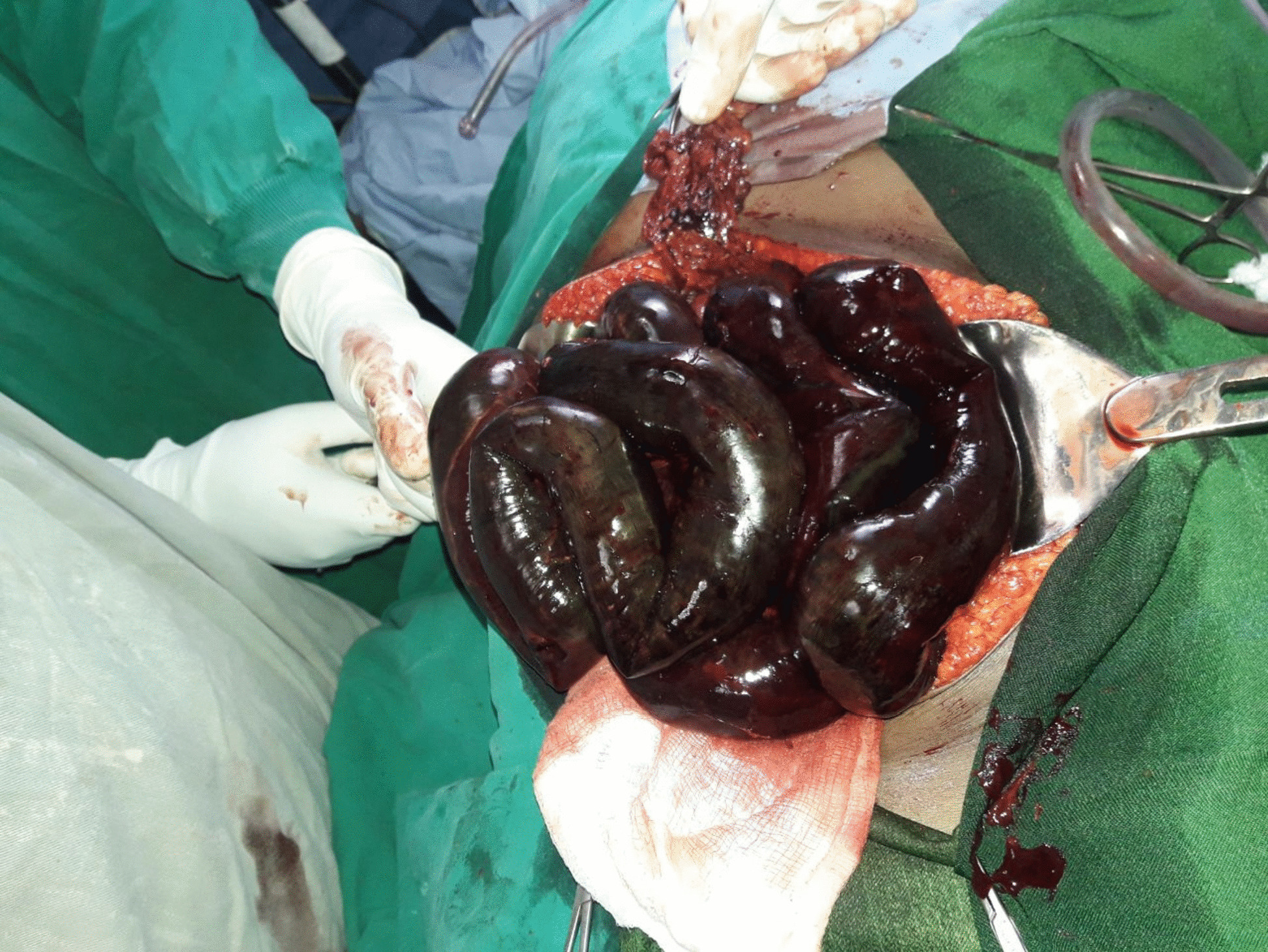
intraoperative picture showing gangrenous small bowel

**Fig. 3:**
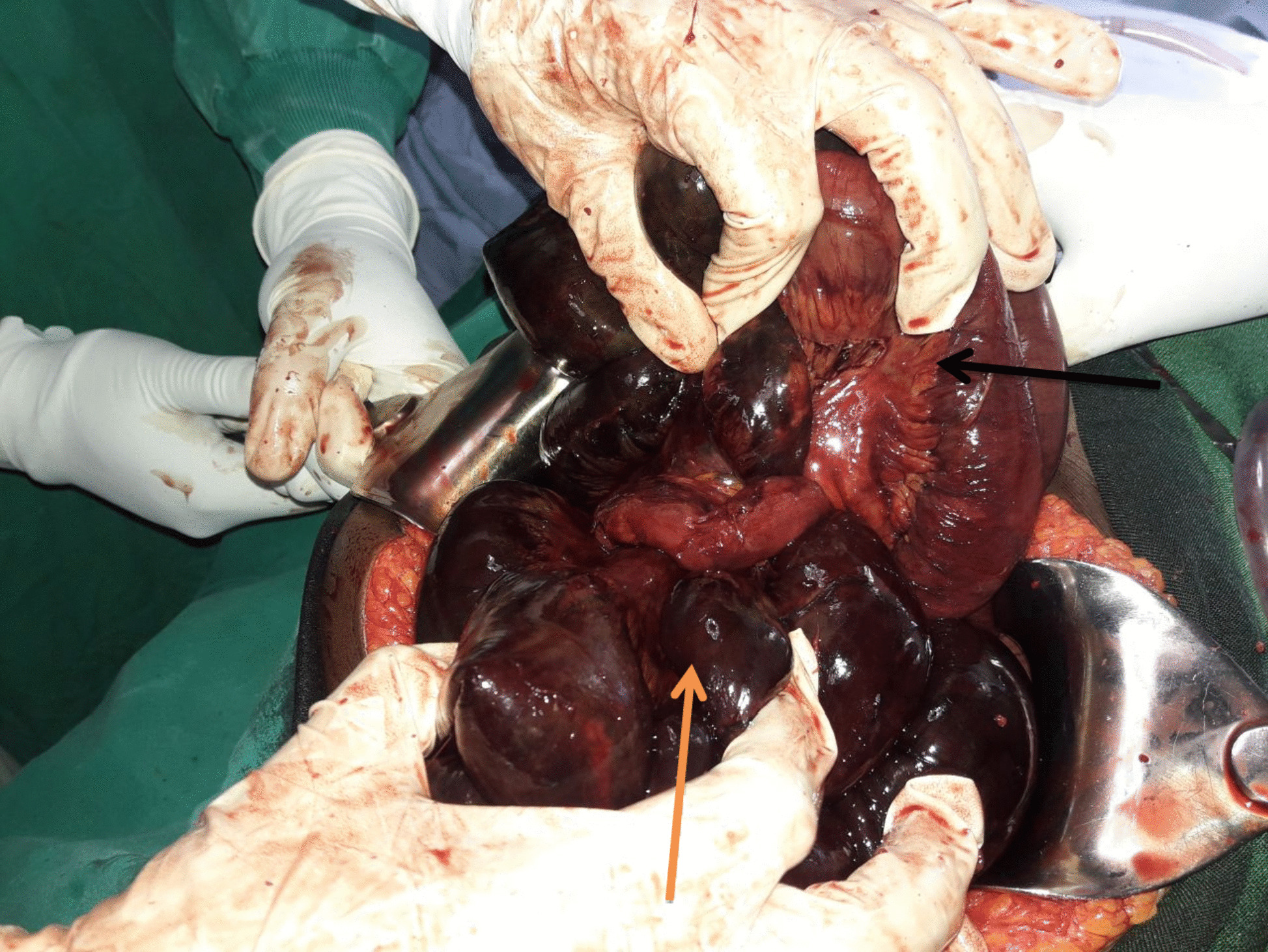
Intraoperative picture showing loop of the small intestine making a knot on the distal ileal segment resulting in gangrene of most of the loops (the pink arrow was the distal segment and the black segment was the proximal segment)

With a diagnosis of acute small bowel obstruction with peritonitis secondary to gangrenous small bowel volvulus, she was resuscitated with normal saline, an NG tube was inserted, and surgery was offered. However, the patient refused surgery, and the surgery was delayed for 13 h. Later, the patient agreed to surgery. Intraoperatively, there was 300 ml of reactive fluid in the peritoneal cavity, and there was frankly gangrenous small bowel with ileo-ileal knotting (Figs. 2 and 3). The whole small bowel is gangrenous except for the proximal 90 cm of the jejunum and the distal 6 cm of the ileum. The viable small bowel was grossly edematous. Other abdominal viscera were grossly normal. What was done was: reactive fluid sucked, gangrenous small bowel resected, distal ileum closed in two layers, and proximal jejunum exteriorized as an end stoma. The abdominal cavity was washed with warm normal saline, and the abdomen was closed in layers and the skin left open for wound care.

Subsequently, she was started on ceftriaxone 1 mg IV twice a day, metronidazole 500 mg IV three times a day, and analgesics. Relaparotomy was done after four days; during this time, restorative jejuno-ileal anastomosis was done, and the wound was closed with a retention suture. The patient’s initial course was rough and complicated by hypokalemia, hypoalbuminemia, and sepsis, which were treated by IV KCL, albumin infusion, and antibiotics. She made a remarkable recovery after that and was discharged after 12 days of hospital stay. At discharge, the patient was advised to eat small, frequent meals with high protein and low simple sugars, such as peanut butter, oatmeal, and barley. She was also advised to minimize liquid between meals and increase soluble-fiber intake (Ethiopian food is highly fiber-rich). On subsequent follow-up at the surgical referral clinic at 2 weeks, 1 month, and 3 months, her body weight was progressively increasing, and she was tolerating meals well.

## Discussion

Small bowel obstruction is the leading cause of hospital admission to the surgical ward and is a major cause of morbidity and financial expenditure across the globe. Adhesion is the leading cause of small bowel obstruction in the western world [1]. However, according to a systematic review done in Ethiopia, small bowel volvulus is the leading cause followed by intussusception and adhesion. Overall, among 755 patients with small bowel obstruction, only one of them had ileo-ileal knotting [5]. Intestinal knot is an uncommon cause of strangulated intestinal obstruction. It is the obstruction of an intestinal segment exhibiting the closed loop phenomenon because of a knot of the mesentery. There have been reports of several intestinal knot formation types, including ileo-caecal, ileo-ileal, ceco-sigmoid, and ileo-ileal. [6, 7]. Among them, the ileo-ileal knot is a very rare entity [7]. In both developed and developing nations, ileo-ileal knotting is rarely discussed in the literature. Few cases of ileo-ileal knotting have been reported in Ethiopia, as far as we are aware [7, 8]. Age or sex does not appear to be a significant factor in the cases that have been documented in individuals as young as 11 months old to as old as 80 years old. Though reports of the entity are more numerous in Eastern Europe, Asia, and Africa, they are less common in the West for some reason [9].

Ileo-ileal knotting is a very rare cause of small bowel obstruction and it results in rapid gangrene of the affected bowel segment. The cause of ileo-ileal knotting is not known. however, freely mobile intestine and redundant sigmoid colon coupled with long and narrow mesentery have been postulated to be a risk factor for ileo-sigmoid knotting may contribute to it as well [4]. A high fiber bulky diet and excessive mobility of the ileum may be related to ileo-ileal knotting. Ileo-ileal knotting has a high mortality rate and it is around 50% [10]. The literature describes significant rates of morbidities and mortality in addition to gangrene, which occurred at a rate of 78–80% [9, 11, 12].

The preoperative diagnosis of ileo-ileal knotting is very difficult or impossible and it is usually diagnosed intraoperatively as there are no specific etiologic, clinical, and radiologic features, these patients usually present with signs and symptoms of intestinal obstruction with rapid progress and deterioration. One report showed that CT findings help in the preoperative diagnosis of intestinal knotting [13]. The CT scan finding include a whirl created by the twisted intestine and sigmoid mesocolon, and a radial distribution of the intestine and mesenteric vascular structures [14]. The management of it depends on the viability of the affected segment, if viable, untying the knot is recommended. However, if the bowel is not viable like our patient en bloc resection followed by anastomosis or exteriorization is preferred to avoid perforation and further contamination [15].

Whenever the diagnosis of ileo-ileal knotting is suspected, treatment should be started as soon as possible which includes aggressive IV fluid resuscitation, insertion of a nasogastric tube and initiation of broad-spectrum antibiotics. Once the patient is adequately resuscitated, an emergency laparotomy should be performed. The type of operation depends on the viability of the affected segment. Different recommendations exist for the management of ileoileal knotting. To prevent unnecessary bowel resection, some recommend releasing the knot first in order to assess the amount of salvageable small bowel. On the other hand, some argue against releasing the knot in order to avoid contaminating the surgical site and allowing necrotic material to enter the bloodstream [16].

Since recurrence is uncommon if the bowel is still viable and strangulation has not yet taken control, untwisting the knot alone is generally indicated [15]. However, perforation is a possibility, especially if several attempts are made. En bloc resection of the gangrenous segment following controlled decompression of its contents by enterotomy followed by exteriorization or anastomosis, depending on the surgeon's choice and in the best interest of the patient, is preferred if the bowel is nonviable [17]. In addition, the patient's general condition will determine whether or not it is necessary to undergo primary anastomosis to restore gastrointestinal continuity. Most individuals who have stable hemodynamics have primary anastomosis. A stoma is used to manage some patients who are persistently in shock or have severely edematous intestines [18].

Postoperatively patient should be monitored for hydration status, nutritional status, electrolyte disturbances, anemia, and signs of anastomotic leak (If an anastomosis is performed). Depending on the length of the remaining small bowel, follow-up should include signs of short bowel syndrome and malnutrition. If that happens, management should be started as early as possible starting with dietary modification. The Ileocecal valve should be preserved whenever possible as it reduces the risk of short bowel syndrome [2, 7].

## Conclusion

Ileo-ileal knotting is a very rare entity. However, it should always be considered in the differential diagnosis of strangulated small bowel obstruction as it is associated with high mortality and morbidity. A high index of suspicion coupled with early surgical intervention has paramount importance in the management of this unusual but deadly condition.

## Data Availability

The authors of this manuscript are willing to provide additional information regarding the case report.

## Abbreviations

IV: Intravenous
NG tube: Naso-gastric tube
SBO: Small bowel obstruction
